# Hemofilters versus hemodialyzers: impacts on cytokine removal during cardiopulmonary bypass in pediatric cardiac surgery: a randomized controlled trial

**DOI:** 10.1186/s13019-026-04058-0

**Published:** 2026-04-13

**Authors:** Ahmed M. Abdelazim, Basma Alaaeldin, Akram M. Amer, Amal Rizk, Dina Mahmoud, Yasmin Elsobky, Hadeel Tawfik, Ahmed S. Fadaly, Mahmoud Elshazly

**Affiliations:** 1 Cardiothoracic surgery Department, Alnas Hospital, Shubra El Kheima, Egypt; 2 Department of Anesthesia, Intensive Care & Pain Management, Ain Shams University, Cairo, Egypt; 3 Anesthesia Department, Alnas hospital, Shubra El Kheima, Egypt; 4 Critical Care Department, Cairo University, Cairo, Egypt; 5 Former Laboratory and Blood Bank Director, Alnas Academy Medical Program Director, Alnas hospital, Shubra El Kheima, Egypt; 6 Medical Laboratory Specialist, Laboratory and Blood Bank Department, Alnas hospital, Shubra El Kheima, Egypt; 7 Deputy Director, Alnas Clinical Research Center (CRC), Alnas Hospital, Shubra El Kheima, Egypt; 8 Biostatistician, Alnas Clinical Research Center (CRC), Alnas hospital, Shubra El Kheima, Egypt; 9 Cardiothoracic Surgery Department, Faculty of Human medicine, Zagazig University, Zagazig, Egypt; 10 Cardiothoracic Surgery Department, Cairo University Hospitals, Cairo, Egypt

**Keywords:** Congenital heart disease, Cardiopulmonary bypass, Zero-balance ultrafiltration, Hemodialyzer, Hemofilter, Cytokines, Pediatric cardiac surgery

## Abstract

**Background:**

Congenital heart disease (CHD) remains a leading cause of pediatric morbidity and mortality worldwide. Cardiopulmonary bypass (CPB) is crucial for surgical repair but is associated with systemic inflammation and/or end-organ dysfunction. Ultrafiltration (UF) techniques, particularly zero-balance ultrafiltration (Z-BUF), have been developed to mitigate these effects by removing cytokines. Conventional hemofilters are widely used, but in low- and middle-income countries, high-flux hemodialyzers are increasingly substituted, although evidence for their equivalence in pediatric CPB remains scarce.

**Methods:**

In this prospective, single-blinded, randomized controlled noninferiority trial, a total of 60 pediatric patients (aged 1–15 years) who underwent elective CPB for congenital heart surgery were enrolled. Thirty patients were assigned to the Medica hemofilter group, and thirty to the Fresenius Helixone^®^ hemodialyzer group. Interleukin-6 (IL-6) was designated as the primary endpoint, whereas interleukin-1β (IL-1β), hematocrit, lactate levels, mechanical ventilation (MV) duration, length of stay in the intensive care unit (ICU), and overall hospital stay served as secondary endpoints. Cytokine levels were measured at baseline (T1), before Z-BUF (T2), and immediately after Z-BUF (T3). Prespecified noninferiority margins were applied, and generalized estimating equations (GEEs) were used to assess temporal trends.

**Results:**

Baseline characteristics and procedural complexity were comparable between cohorts. Following Z-BUF, changes in IL-1β (median difference − 0.223 pg/mL) and IL-6 (0.642 pg/mL) met the predefined noninferiority criteria (*p* < 0.001 for both). GEE analysis revealed no significant interaction between filter type and cytokine trends. The median hematocrit, lactate levels, MV duration, length of stay in the ICU, and overall hospital stay were also similar between the study arms. Mortality was identical (3.3%) in both arms. Importantly, no cases of acute kidney injury or acute neurological events were observed in either group.

**Conclusions:**

Compared with conventional hemofilters, high-flux hemodialyzers are noninferior in controlling intraoperative cytokines during Z-BUF in pediatric CPB and demonstrate comparable clinical safety outcomes. Hemodialyzers appear to be an effective and practical alternative in resource-limited settings. Validation in larger multicenter trials with extended postoperative sampling is warranted.

**Trial registration and date:**

ClinicalTrials.gov (NCT06792565) on 22 March 2024.

## Background

Congenital heart disease (CHD) remains the most common congenital anomaly and a major contributor to childhood morbidity and mortality worldwide [1]. It affects nearly 1.8% of live births, with approximately half a million children in Africa alone requiring specialized cardiological care each year [2, 3]. The introduction of cardiopulmonary bypass (CPB) by Gibbon Jr. in 1953 revolutionized the treatment of CHD, enabling safe intracardiac repair of complex malformations [4]. In pediatric cardiac surgery, CPB has become indispensable, although it poses unique challenges related to the small circulating blood volume and immature organ systems of pediatric patients [5].

Despite its life-saving role, CPB induces profound inflammation, fluid shifts, and end-organ dysfunction, particularly in pediatric patients [6]. This response may intensify to systemic inflammatory response syndrome (SIRS), which occurs in up to one-third of children after congenital heart surgery, especially those of low age, low body weight, or prolonged bypass duration [7, 8]. CPB-associated SIRS results from complement activation and proinflammatory cytokine release, which together promote endothelial injury, neutrophil activation, and organ dysfunction [9]. Ultrafiltration (UF) has therefore become a key mechanical strategy to attenuate CPB-related inflammation by removing plasma water and low-molecular-weight solutes, thereby reducing cytokine burden and preventing fluid overload [10, 11].

Among UF modalities, zero-balance ultrafiltration (Z-BUF), first introduced by Journois et al. in the pediatric population, aims to reduce the inflammatory mediator load during the rewarming phase by exchanging plasma water with crystalloids while filtering solutes up to approximately 66 kilodaltons (kDa) [12, 13]. This technique attenuates cytokine accumulation, improves cardiopulmonary recovery, and corrects electrolyte and acid-base imbalances; however, cytokine clearance remains variable and is influenced by protein binding and membrane characteristics [5, 11, 14]. Early reports in adults and children described the integration of dialysis membranes during CPB to manage solute balance and fluid removal, confirming technical feasibility but without specific cytokine analysis [15–17].

In many low- and middle-income countries (LMICs), including Egypt, financial constraints limit access to hemofilters, prompting perfusionists to use hemodialyzers as alternatives during CPB. While preliminary data suggest comparable safety, evidence regarding their efficacy for cytokine removal remains limited. Given these challenges, this study aimed to evaluate whether high-flux hemodialyzers are noninferior to conventional hemofilters in controlling cytokine levels during Z-BUF in pediatric CPB, with the goal of identifying a safe and practical alternative for resource-limited settings. We hypothesized that the use of a high-flux hemodialyzer would achieve cytokine removal comparable to that of a standard hemofilter without compromising clinical safety or postoperative outcomes.

## Methods and materials

### Study design and population

This prospective, single-blinded, randomized controlled trial was conducted at Alnas Hospital, Qalyubia, Egypt, between March 2024 and June 2025. Sixty pediatric patients who underwent elective CPB for congenital heart surgery were enrolled and randomly assigned to either the hemofilter group (*n* = 30) or the hemodialyzer group (*n* = 30) using computer-generated block randomization with variable block sizes of four and eight. Allocation concealment was ensured, and blinding was maintained at the level of patients and their parents/ legal guardians. Due to the nature of the intervention, perfusionists (2 only) could not be blinded; however, perioperative management, UF protocols, laboratory analyses (with analyst blinded to group allocation), and postoperative care were standardized to minimize potential bias.

### Inclusion and exclusion criteria

Eligible patients were pediatric individuals aged 1–15 years who were scheduled for elective cardiothoracic surgery requiring CPB with an expected CPB duration of > 60 min. Patients were excluded if they had preoperative sepsis, prior cardiothoracic surgery, preoperative renal impairment, cardiogenic shock requiring inotropes, or a preoperative lactate level > 2 mmol/L.

### Sample size

Assuming that interleukin (IL) -6 values were normally distributed with an expected population standard deviation of approximately 140 pg/mL, a conservative noninferiority margin was set at 54.2 pg/mL, corresponding to 20% of the historical mean [18] IL-6 concentration reported in pediatric CPB studies by Madhok et al. [19]. Using a significance level (alpha) of 0.05 and study power of 80%, sample size estimation for a continuous outcome noninferiority trial required 21 patients per group as calculated by the Sample Size Calculator from *riskcalc.org* [20]. To account for potential data loss and improve robustness, the sample size was increased to 30 patients per group.

### Surgical and perfusion techniques

All procedures were performed using a Stockert S5^®^ heart–lung machine (LivaNova Deutschland GmbH, München, Germany). The extracorporeal circuit included medical-grade polyvinyl chloride tubing and silicone pump tubing in the roller pump, with a Terumo Capiox membrane oxygenator (Terumo Corporation, Tokyo, Japan) employed in every case. The priming solution consisted of Ringer’s acetate, heparin (7 IU/mL), 5% albumin, and 20% mannitol (0.5 g/kg), with packed red blood cells added when required according to hemoglobin levels. Myocardial protection was achieved with Bretschneider’s histidine–tryptophan–ketoglutarate (HTK^®^) cardioplegia (30 mL/kg). The surgeon suctioned the cardioplegia directly from the coronary sinus, outside the cardiotomy reservoir, after right atrial opening to minimize hemodilution. Surgery was performed under moderate hypothermia (32 °C). Rewarming began immediately after aortic cross-clamp removal, with blood gases managed via an alpha-stat strategy.

### UF technique

Z-BUF was performed in all patients, using 500 mL of Ringer’s acetate once body temperature reached 35 °C following aortic cross-clamp removal. No Conventional UF was applied intraoperatively during the surgery. Patients were randomized into two groups: the hemofilter group, which received the Medica DP 03 HC PUREMA polyethersulfone hemofilter (Medica, Medolla, Italy), and the hemodialyzer group, which received a Fresenius Helixone^®^ high-flux hemodialyzer (Fresenius Medical Care, Bad Homburg, Germany), selected according to body weight (FX Paed for patients < 10 kg; FX50 for patients ≥ 10 kg).

### Sampling and laboratory analysis

Blood samples were collected via the peripheral arterial line at three time points:

T1: After induction of anesthesia and before the skin incision.

T2: Immediately before Z-BUF initiation.

T3: Immediately after Z-BUF completion.

Serum concentrations of IL-1β and IL-6 were measured using the Quantikine Enzyme-Linked Immunosorbent Assay (ELISA) Kit (R&D Systems, Inc., Minneapolis, MN, USA) and analyzed using the ELISA sandwich technique with monoclonal antibodies on a Robonik Readwell Touch ELISA Microplate Analyzer (Robonik India Pvt. Ltd., Navi Mumbai, India). Hematocrit levels (before and after Z-BUF) and lactate concentrations (after Z-BUF) were measured using an ABL-800 FLEX Blood Gas Analyzer (Radiometer Medical, Brønshøj, Denmark).

### Data collection and outcomes

Data were prospectively collected using a structured case record form that included demographic characteristics (age, sex, weight, height, and body surface area), operative and perfusion data (surgical type, CPB duration, aortic cross-clamp duration, and risk adjustment in congenital heart surgery (RACHS-1) score, laboratory results (IL-1β, IL-6, hematocrit, and lactate), and postoperative outcomes (mechanical ventilation (MV) duration, intensive care unit (ICU) length of stay (LOS) and overall hospital stay). The primary outcome of the study was the change in the IL-6 concentration as a marker of systemic inflammation, while the secondary outcomes included the IL-1β concentration, hematocrit and lactate levels, MV duration, ICU LOS, and overall hospital stay.

### Informed consent and ethical considerations

The study protocol was approved by the Institutional Review Board of Alnas Hospital (Approval Number: RHDIRB-NA-240823-01UC-NPON0323-0002) on 25 February 2024 and was registered at ClinicalTrials.gov (Identifier: NCT06792565) on 22 March 2024. Written informed consent was obtained from the parents/ legal guardians prior to surgery. Patient confidentiality was strictly maintained in accordance with institutional policies, with all records coded and securely stored. Access to the data was restricted to the study team, and all investigators signed a confidentiality agreement.

### Statistical analysis

Continuous variables that were not normally distributed, as assessed by the Shapiro–Wilk test, were summarized as medians with interquartile ranges (IQRs). Categorical variables are reported as frequency counts and percentages in the results. Given the nonnormality of the continuous data, all analyses involving these variables were conducted using appropriate nonparametric statistical methods.

The noninferiority of the hemodialyzer over the hemofilter was assessed for serum IL-1β and IL-6 levels after Z-BUF (T3). The noninferiority margin for each cytokine was set at 20% of its historical mean concentration [18], 24.2 pg/mL for IL-1β and 54.2 pg/mL for IL-6, on the basis of the values reported by Madhok et al. [19]. A one-sided Wilcoxon rank-sum test was used to evaluate whether the difference was greater than the negative margin. The 95% confidence intervals (CIs) were derived to test the hypotheses.

We used generalized estimating equations (GEEs) to analyze IL-1β and IL-6 levels over time. The model accounted for repeated measures within subjects using an exchangeable correlation structure. The effects of time point, filter type (hemodialyzer versus hemofilter), and their interactions were assessed. This interaction term specifically tests whether the pattern of change over time differs between the two types of filters.

All the statistical analyses were performed using R software (version 4.5.1) and RStudio [21, 22]. The primary analysis was a test of noninferiority using a one-sided alpha level of 0.025 and the prespecified margin described above. Secondary outcomes were tested for differences using a two-sided alpha level of 0.05. GEE models were implemented using the geepack package in R [23].

## Results

Baseline demographic characteristics, surgical risk profiles, and operative diagnoses were comparable between the hemodialyzer and hemofilter groups. The majority of patients in both groups fell within lower RACHS-1 categories, and common procedures included repairs for Tetralogy of Fallot (TOF) and ventricular septal defects, as demonstrated in Table 1.

Analysis of the perioperative and outcome data revealed no statistically significant differences between the hemodialyzer and hemofilter study arms. The median CPB duration and median aortic cross-clamp duration were closely matched, indicating similar procedural complexity. Postoperative outcomes were likewise comparable, with no significant differences in the median duration of MV, median LOS in the ICU, or median overall hospital stay. Mortality was identical, with one death in each arm. After adjusting for multiple comparisons, all the results remained nonsignificant, as shown in Table 2.

For IL-1β, the estimated median difference (hemodialyzer minus hemofilter) was − 0.223 pg/mL. The lower bound of the 95% CI for this difference was − 0.221 pg/mL. This lower bound is the most conservative estimate of the difference between devices and is decisively greater than the negative noninferiority margin of 24.2 pg/mL. This result confirmed the noninferiority of the hemodialyzer (*p* < 0.001). The findings for IL-6 were also consistent. The estimated median difference was 0.642 pg/mL, with a lower 95% CI of -0.716 pg/mL. This lower bound was also significantly above the permitted margin of 54.2 pg/mL, providing strong evidence of noninferiority (*p* < 0.001). In conclusion, on the basis of the post-Z-BUF cytokine levels, the hemodialyzer is statistically noninferior to the hemofilter for both IL-1β and IL-6 as presented in Table 3.

**Fig. 1:**
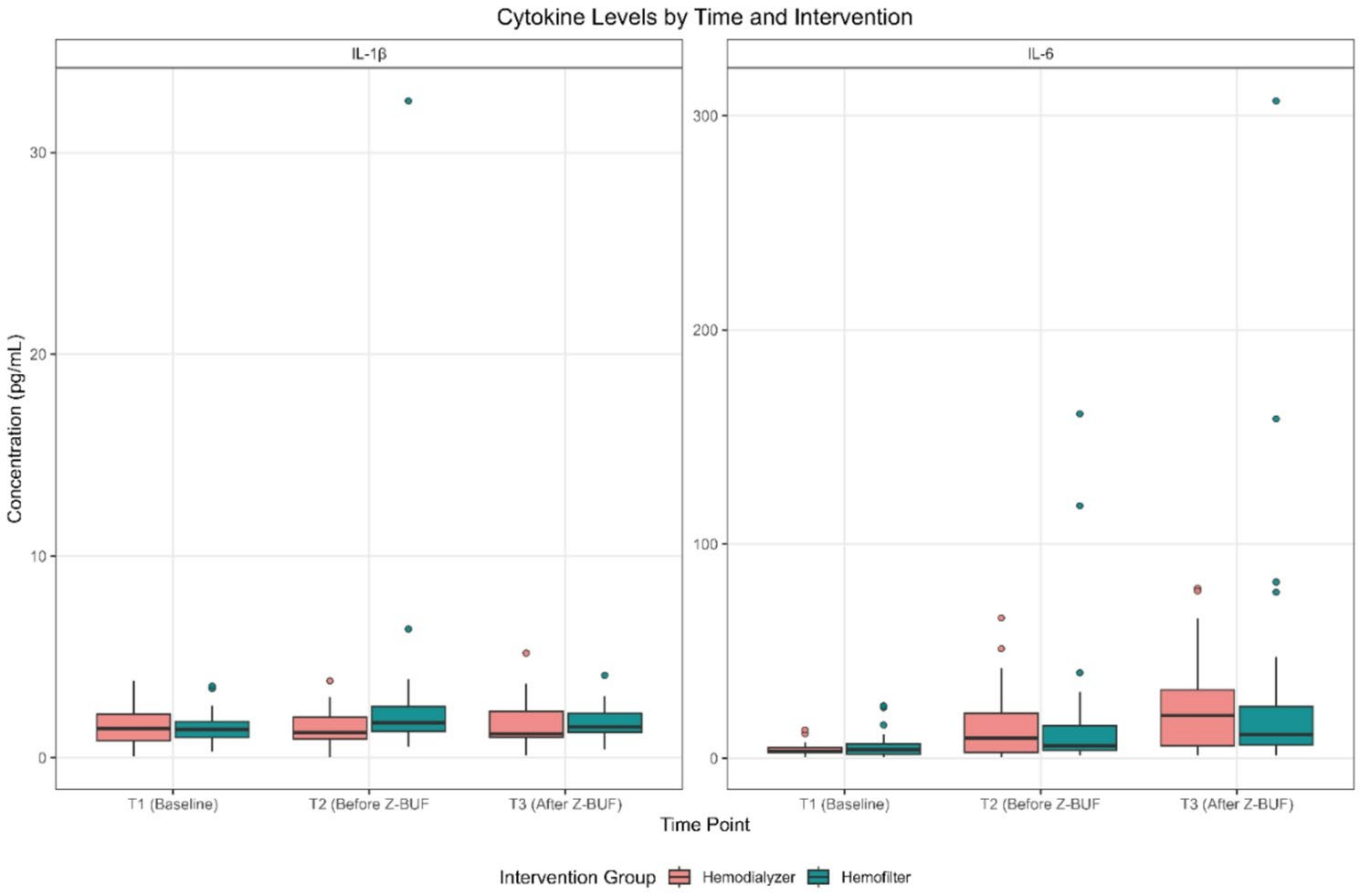
Shows the cytokine levels by time and intervention. Z-BUF indicates Zero-balance ultrafiltration, IL interleukin

**Fig. 2:**
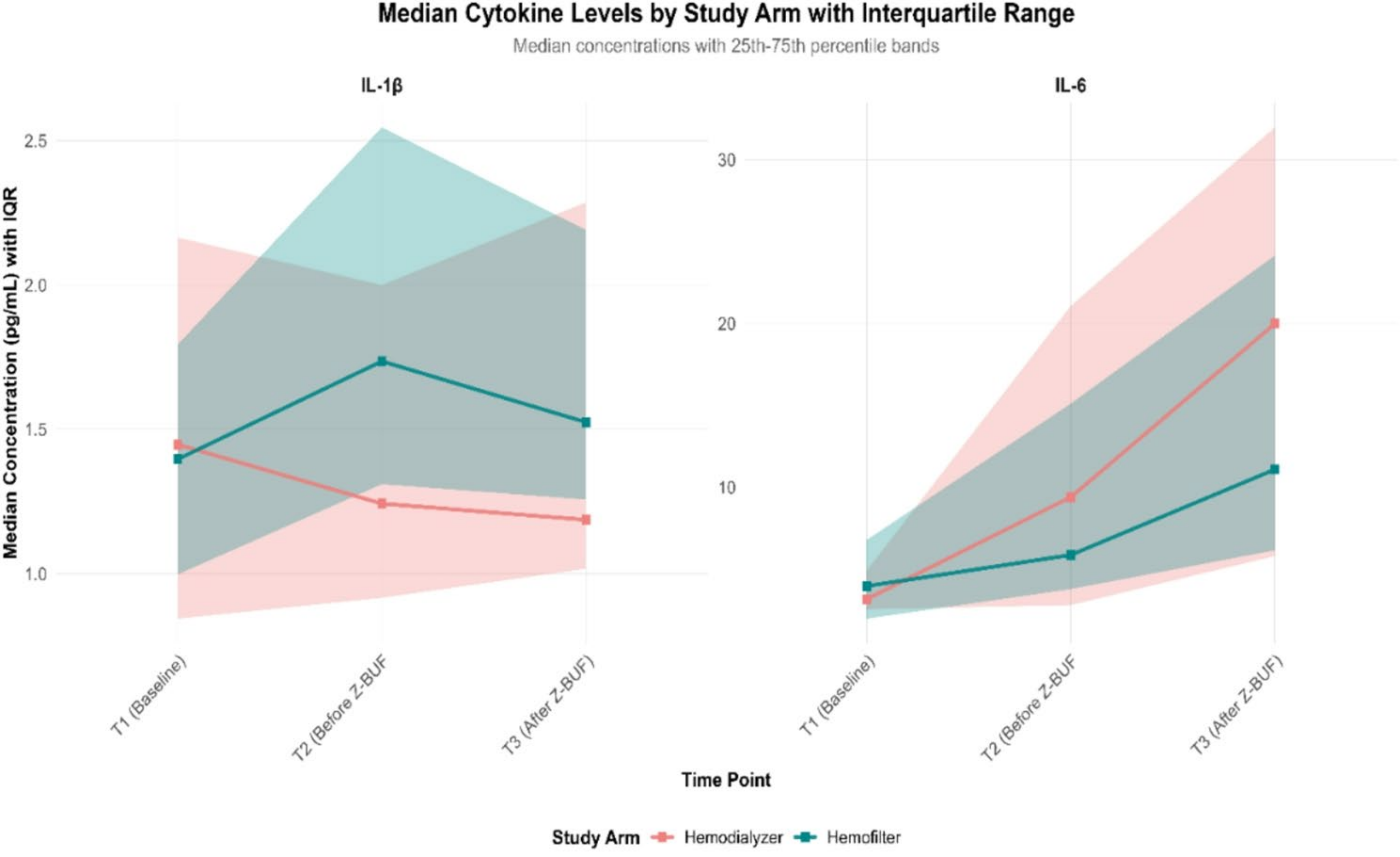
Shows the median cytokine levels by study arm with IQR. Z-BUF indicates Zero-balance ultrafiltration, IL interleukin

To further assess the inflammatory response, cytokine concentrations were evaluated in GEE models. The analysis revealed no statistically significant differences in the IL-1β levels at the final measured time point (after Z-BUF, T3) between the hemofilter and hemodialyzer arms. Specifically, the interaction term between T3 and filter type was not significant (estimate = 0.09, *p* = 0.77), indicating that the cytokine levels measured after UF were similar regardless of the device used. A comparable pattern was observed for IL-6. Compared with those at baseline, levels significantly increased over time in both arms, with estimated increases of 11.21 units at T2 (*p* < 0.001) and 19.37 units at T3 (*p* < 0.001) compared with baseline. However, no statistically significant differences were detected between the hemofilter and hemodialyzer groups, as the interaction term for T3 and filter type was not significant (estimate = 8.28, *p* = 0.47). Taken together, these findings suggest that hemodialyzers demonstrate promising efficacy, comparable to that of conventional hemofilters, in managing cytokine clearance during CPB-associated UF, as shown in Table 3; Figs. 1 and 2.

**Table 1:** Baseline Characteristics by Intervention Groups

Variable	Overall *N* = 60^1^	Hemodialyzer *N* = 30^1^	Hemofilter *N* = 30^1^
Age (years)	2.0 (2.3)	2.0 (2.8)	2.0 (2.0)
Weight (kg)	10.6 (5.6)	10.3 (7.9)	11.1 (4.8)
Height (cm)	83.5 (21.5)	83.0 (18.8)	86.0 (22.5)
BSA (m²)	0.5 (0.2)	0.5 (0.2)	0.5 (0.2)
**Gender**			
Female	22 (37%)	10 (33%)	12 (40%)
Male	38 (63%)	20 (67%)	18 (60%)
**RACHS-1 Category**			
1	4 (6.7%)	2 (6.7%)	2 (6.7%)
2	48 (80%)	26 (87%)	22 (73%)
3	7 (12%)	1 (3.3%)	6 (20%)
4	1 (1.7%)	1 (3.3%)	0 (0%)
**Surgical Procedure**			
Aortic Sub-valvular Repair	1 (1.7%)	1 (3.3%)	0 (0%)
Aortic Valve Repair	1 (1.7%)	0 (0%)	1 (3.3%)
ASD Closure	1 (1.7%)	0 (0%)	1 (3.3%)
ASD Closure + Pulmonary Valvotomy	1 (1.7%)	1 (3.3%)	0 (0%)
ASD Closure + VSD Closure + RVOT Resection	1 (1.7%)	0 (0%)	1 (3.3%)
Complete AVC Repair	2 (3.3%)	0 (0%)	2 (6.7%)
Ebstein Anomaly (Cone Procedure)	1 (1.7%)	1 (3.3%)	0 (0%)
Mitral Valve Repair	1 (1.7%)	0 (0%)	1 (3.3%)
PAPVD Repair	3 (5.0%)	2 (6.7%)	1 (3.3%)
SAM Resection	2 (3.3%)	2 (6.7%)	0 (0%)
TAPVD Repair	1 (1.7%)	0 (0%)	1 (3.3%)
TOF Repair	16 (27%)	11 (37%)	5 (17%)
Transitional AVC Repair	4 (6.7%)	1 (3.3%)	3 (10%)
VSD Closure	10 (17%)	6 (20%)	4 (13%)
VSD Closure + Pulmonary Valvotomy	1 (1.7%)	0 (0%)	1 (3.3%)
VSD Closure + RVOT Resection	6 (10%)	1 (3.3%)	5 (17%)
VSD Closure + SAM Resection	7 (12%)	4 (13%)	3 (10%)
VSD Closure + SAM Resection + RVOT Resection	1 (1.7%)	0 (0%)	1 (3.3%)

^1^ Median (IQR); n (%). BSA indicates Body Surface Area, RACHS-1 Risk Adjustment for Congenital Heart Surgery, ASD Atrial Septal Defect, VSD Ventricular Septal Defect, RVOT Right Ventricle Outflow Tract, AVC Atrio-ventricular Canal, PAPVD Partial Anomaly Pulmonary Venous Drainage, SAM Sub-aortic Membrane, TAPVD Total Anomaly Pulmonary Venous Drainage, TOF Tetralogy of Fallot

**Table 2:** Surgery-Related and Outcome Variables by Intervention Groups

Variable^1^	Hemodialyzer *N* = 30^2^	Hemofilter *N* = 30^2^	*p*-value ^3^
CPB Duration (minutes)	90.0 (43.0)	91.0 (48.0)	0.906
Aortic Cross-Clamp Time (minutes)	58.5 (35.0)	61.5 (29.5)	0.491
MV Duration (hours)	6.0 (12.8)	5.0 (15.8)	0.783
LOS in ICU (days)	2.0 (1.8)	2.0 (1.8)	0.975
Overall Hospital Stay (days)	7.0 (3.8)	7.0 (3.8)	0.726
Mortality	1 (3.3%)	1 (3.3%)	> 0.999

^1^ Statistical test: Continuous variables analyzed with Wilcoxon rank-sum test; categorical variables analyzed with Fisher’s exact test

^2^ Median (IQR); n (%)

3 p-values represent raw values; all remained non-significant (*p* > 0.05) after Benjamini-Hochberg adjustment for multiple comparisons. CPB indicates Cardiopulmonary Bypass, MV Mechanical Ventilation, LOS Length of Stay, ICU Intensive Care Unit

**Table 3:** Comparison of Biochemical Markers across Intervention Groups

Variable^1^	Hemodialyzer *N* = 30^2^	Hemofilter *N* = 30^2^	*p*-value ^3^
Hematocrit Before Z-BUF (%)	30.50 (4.50)	32.00 (3.00)	0.109
Hematocrit After Z-BUF (%)	33.00 (4.75)	33.00 (3.00)	0.776
Lactate After Z-BUF (mg/dL)	1.90 (0.83)	2.00 (0.98)	0.678
IL-1β at T1 (Baseline), pg/mL	1.45 (1.32)	1.40 (0.80)	0.971
IL-1β at T2 (Before Z-BUF), pg/mL	1.24 (1.08)	1.74 (1.24)	0.04
IL-1β at T3 (After Z-BUF), pg/mL	1.19 (1.27)	1.53 (0.93)	0.297
IL-6 at T1 (Baseline), pg/mL	3.17 (2.36)	3.96 (4.80)	0.371
IL-6 at T2 (Before Z-BUF), pg/mL	9.40 (18.26)	5.86 (11.33)	0.888
IL-6 at T3 (After Z-BUF), pg/mL	19.99 (26.18)	11.09 (17.99)	0.877
Δ IL-1β (T2 - T1), pg/mL	-0.07 (0.92)	0.23 (1.69)	0.212
Δ IL-1β (T3 - T1), pg/mL	-0.24 (1.82)	0.24 (1.40)	0.723
Δ IL-6 (T2 - T1), pg/mL	5.60 (17.14)	2.68 (7.23)	0.363
Δ IL-6 (T3 - T1), pg/mL	14.64 (24.54)	6.55 (15.18)	0.53
% Change IL-1β (T2 vs. T1)	-4.10 (61.63)	15.77 (151.92)	0.34
% Change IL-1β (T3 vs. T1)	-16.55 (197.63)	19.10 (113.94)	0.589
% Change IL-6 (T2 vs. T1)	182.87 (281.42)	136.86 (219.20)	0.6
% Change IL-6 (T3 vs. T1)	253.59 (594.82)	208.72 (426.32)	0.6

^1^ Statistical test: Wilcoxon rank-sum test for all biochemical variables

^2^ Median (IQR)

^3^ p-values represent raw values; all remained non-significant (*p* > 0.05) after Benjamini-Hochberg adjustment for multiple comparisons. Z-BUF indicates Zero-Balanced Ultrafiltration, IL interleukin

## Discussion

In resource-limited settings, the use of hemodialyzers as an alternative to hemofilters has emerged as a practical solution [24]. Fresenius Helixone^®^ high-flux hemodialyzers, with pediatric-suited surface areas (0.2–1.0 m²), offer effective solute clearance while minimizing the risk of hemodynamic instability [25]. Prior studies in critical care have shown that high cut-off hemodialyzers can remove proinflammatory cytokines during renal replacement therapies, supporting their potential utility in CPB [26, 27]. Their adoption in pediatric cardiac surgery not only provides comparable inflammatory control and fluid balance but also facilitates earlier extubation and reduces pulmonary complications, with safety outcomes in line with those of conventional hemofilters [24].

### Comparison with other studies

This study demonstrated that the use of hemodialyzer was statistically noninferior to the use of hemofilter for removing IL-1β and IL-6 during Z-BUF in pediatric patients undergoing cardiac surgery. The noninferiority analysis revealed that the median differences for IL-1β (− 0.223 pg/mL) and IL-6 (0.642 pg/mL) were well within the predefined noninferiority margins of 24.2 pg/mL and 54.2 pg/mL, respectively, providing robust evidence that hemodialyzers are comparable to conventional hemofilters in attenuating the release of inflammatory cytokines. These findings are clinically meaningful, given that both IL-1β (17 kDa) and IL-6 (26 kDa) fall well below the molecular weight cut-off of high-flux hemodialyzers and hemofilters (~ 30–50 kDa), suggesting that the observed equivalence in cytokine clearance is physiologically expected [28, 29].

Furthermore, the GEE analysis confirmed that IL-6 levels increased significantly over time in both allocation arms, whereas IL-1β levels showed no significant temporal differences, findings that are consistent with cytokine release patterns reported in prior pediatric CPB studies [11, 30]. This postoperative rise in IL-6 reflects the well-established inflammatory response induced by CPB. UF strategies such as Z-BUF are intended to modulate and attenuate cytokine release rather than completely eliminate inflammation; therefore, the observed noninferiority indicates comparable inflammatory control between the devices rather than absence of cytokine activation.

The median clinical outcomes were also comparable between the two study arms. CPB duration, aortic cross-clamp duration, MV duration, ICU LOS, and overall hospital stay were not significantly different. These values are consistent with established benchmarks for pediatric congenital heart surgery and align with the findings reported in previous studies [6, 31]. Lactate values after Z-BUF remained below the hyperlactatemia threshold, which is consistent with favorable postoperative metabolic stability, as reported by Gerami et al. [32]. The hematocrit values achieved post-CPB are in line with present recommendations to enhance oxygen delivery while limiting hemodilution as demonstrated by Ramakrishnan et al. [4]. This consistency across multiple key metrics underscores the successful management of these pediatric cases and provides further support for the clinical approach utilized.

Mortality in our cohort was identical across groups, with one death in each group. Both occurred in patients who underwent TOF repair with a RACHS-1 score of 2, and the causes of death were related to septic and cardiogenic shock rather than device performance. The hemofilter patient demonstrated markedly prolonged CPB duration (244 versus 150 min) and aortic cross-clamp duration (140 versus 76 min), as well as extended MV duration (316 versus 312 h) and ICU LOS (16 versus 12 days) compared with the hemodialyzer patient. Post-Z-BUF cytokine levels (IL-1β: 0.64 versus 2.7 pg/mL; IL-6:22.42 versus 21.17 pg/mL) were comparable and within the range of those of survivors, indicating that mortality was unlikely to be attributable to the UF device. Instead, an extended operative duration and postoperative complications likely contributed more significantly. This finding is consistent with prior literature showing that pediatric cardiac surgical mortality is influenced mainly by operative complexity, CPB duration, and infection risk rather than UF modality [33, 34]. The observed mortality rate of 3.3% per group also aligns with international benchmarks, where congenital heart surgery mortality after CPB ranges from 3.4 to 6.9% [33, 35], supporting the overall safety of both hemodialyzers and hemofilters in this setting.

No cases of acute kidney injury (AKI) or acute neurological events were reported in the hospital records for either group, reinforcing the safety of both the hemodialyzers and hemofilters used in the study. These findings are in agreement with those of Singh et al., who reported no renal complications with hemofiltration during CPB [10]. Similarly, Ramakrishnan et al. reported an overall postoperative AKI incidence of 1.3% with no cases of severe renal failure and reported acute neurological event rates below 1% [4]. Taken together, our results suggest that filter type does not influence early renal or neurological outcomes, and overall, both devices provided comparable hematologic, metabolic, renal, and neurological safety.

Our findings are consistent with those of Abdelazim et al., who demonstrated that pediatric hemodialyzers can be safely and effectively used during CPB, yielding comparable outcomes in terms of hematocrit correction, lactate clearance, and recovery parameters [24]. Similarly, Bierer et al. highlighted that continuous UF modalities, including Z-BUF, attenuate systemic inflammation and improve cardiopulmonary recovery; however, their efficacy appears largely independent of the filter type, provided that the pore size is sufficient for cytokine clearance [11, 14]. In contrast, earlier studies reported significant reductions in IL-6 after CPB when hemofiltration was applied intraoperatively [36] or through Modified UF protocols [37], whereas our data showed persistent postoperative IL-6 elevation, underscoring differences related to UF timing and technique. Notably, Singh et al. also found no intergroup differences in IL-6 removal during pediatric TOF repair [10], further reinforcing our observation of functional comparability between hemodialyzers and hemofilters.

The findings of this noninferiority trial have important implications for resource-limited healthcare systems. In many LMICs, the high cost and inconsistent availability of hemofilters frequently limit the routine use of UF during pediatric CPB. Our demonstration that high-flux hemodialyzers are noninferior to conventional hemofilters for cytokine control during Z-BUF provides a pragmatic and cost-effective alternative that may help sustain pediatric cardiac surgical programs. Hemodialyzers are widely available, familiar to perfusion teams, and substantially less expensive, allowing broader implementation of inflammatory modulation strategies without compromising patient safety or clinical outcomes. Adoption of this approach may reduce procedural costs, prevent surgery delays related to device shortages, and enhance access to advanced perioperative care in constrained settings.

### Study limitations

This study has several limitations. First, its single-center design and relatively small sample size may limit generalizability to other institutions with differing patient populations, perfusion practices, or resource availability, despite the randomized noninferiority design. Second, cytokine measurements were confined to intraoperative timepoints due to financial constraints, precluding evaluation of postoperative inflammatory trajectories. Moreover, only IL-1β and IL-6 were assessed; inclusion of additional mediators such as Tumor Necrosis Factor-α, IL-8, or IL-10 could have provided a more comprehensive characterization of CPB-related inflammation. Third, long-term clinical outcomes, including organ dysfunction, neurodevelopmental status, and rehospitalization, were not evaluated, limiting interpretation beyond the early perioperative period. Finally, clinically validated perioperative cytokine thresholds associated with improved outcomes in pediatric CPB are currently lacking. Consequently, the noninferiority margin was derived from historical cytokine data rather than outcome-based biological targets, reflecting a broader gap in the existing literature rather than a limitation unique to this study.

Despite these limitations, this study represents, to our knowledge, the first randomized noninferiority trial to systematically evaluate the safety and efficacy of hemodialyzers as an alternative to hemofilters during CPB. The findings provide preliminary evidence with particular relevance to LMICs, where cost and device availability are critical considerations, and they support the need for larger, multicenter trials with extended biomarker profiling and long-term follow-up.

## Conclusions

In this randomized controlled noninferiority trial, high-flux hemodialyzers demonstrated noninferior performance compared with conventional hemofilters in modulating IL-1β and IL-6 levels during pediatric CPB with Z-BUF. Clinical outcomes, including hematocrit, lactate levels, duration of MV, ICU and hospital LOS, and in-hospital mortality, were comparable between groups. Within the limits of this single-center study, these findings suggest that high-flux hemodialyzers may represent a feasible and safe alternative to hemofilters, particularly in resource-limited settings where device availability and cost are significant considerations.

## Data Availability

The datasets used and/or analyzed during the current study are available from the corresponding author on reasonable request.

## Abbreviations

CHD: Congenital Heart Disease
CPB: Cardiopulmonary Bypass
SIRS: Systemic Inflammatory Response Syndrome
UF: Ultrafiltration
Z-BUF: Zero-Balance Ultrafiltration
kDa: Kilodaltons
LMICs: low- and middle-income countries
IL: Interleukin
ELISA: Enzyme-Linked Immunosorbent Assay
RACHS-1: Risk Adjustment in Congenital Heart Surgery
MV: Mechanical Ventilation
ICU: Intensive Care Unit
LOS: Length of Stay
IQR: Interquartile Range
CI: Confidence Interval
GEE: Generalized Estimating Equations
TOF: Tetralogy of Fallot
AKI: Acute Kidney Injury
